# Protection against Blast-Induced Traumatic Brain Injury by Increase in Brain Volume

**DOI:** 10.1155/2017/2075463

**Published:** 2017-04-10

**Authors:** Ming Gu, Usmah Kawoos, Richard McCarron, Mikulas Chavko

**Affiliations:** ^1^NeuroTrauma Department, Naval Medical Research Center, Silver Spring, MD, USA; ^2^Department of Surgery, Uniformed Services University of the Health Sciences and the Walter Reed National Military Medical Center, Bethesda, MD, USA

## Abstract

Blast-induced traumatic brain injury (bTBI) is a leading cause of injuries in recent military conflicts and it is responsible for an increased number of civilian casualties by terrorist attacks. bTBI includes a variety of neuropathological changes depending on the intensity of blast overpressure (BOP) such as brain edema, neuronal degeneration, diffuse axonal damage, and vascular dysfunction with neurological manifestations of psychological and cognitive abnormalities. Internal jugular vein (IJV) compression is known to reduce intracranial compliance by causing an increase in brain volume and was shown to reduce brain damage during closed impact-induced TBI. We investigated whether IJV compression can attenuate signs of TBI in rats after exposure to BOP. Animals were exposed to three 110 ± 5 kPa BOPs separated by 30 min intervals. Exposure to BOP resulted in a significant decrease of neuronal nuclei (NeuN) together with upregulation of aquaporin-4 (AQP-4), 3-nitrotyrosine (3-NT), and endothelin 1 receptor A (ETRA) expression in frontal cortex and hippocampus one day following exposures. IJV compression attenuated this BOP-induced increase in 3-NT in cortex and ameliorated the upregulation of AQP-4 in hippocampus. These results suggest that elevated intracranial pressure and intracerebral volume have neuroprotective potential in blast-induced TBI.

## 1. Introduction

Traumatic brain injury (TBI) is of increasing concern in both military and civilian populations due to the long-term health problems and costs related to recovery from such injuries. In particular, blast-induced TBI (bTBI) has been classified as the “signature injury” of recent wars in Iraq and Afghanistan [1–3]. The neurological impairment in bTBI may result from different mechanisms including both a direct shock wave effect on brain and an indirect transfer of the shock wave through blood vessels and cerebrospinal fluid (CSF) to the brain [4–7]. Exposure to blast overpressure (BOP) initiates a cascade of cellular pathological processes in brain including damage to the microvasculature and blood-brain barrier (BBB) integrity, followed by increased BBB permeability [8, 9]. The breakdown of the BBB can result in brain edema and increase in intracranial pressure (ICP), accompanied by activation of secondary brain injury by impairing cerebral perfusion and oxygenation [10]. In particular, activation of oxidative mechanisms and neuroinflammation has been shown to contribute to the neurodegeneration and cell apoptosis in secondary brain injury following bTBI [11, 12].

Current treatment of acute TBI includes surgical intervention and supportive care therapies. Treatment of elevated ICP and optimizing cerebral perfusion are the cornerstones of current therapy [13]. Several attempts to minimize secondary brain injury after TBI by pharmacological intervention or hypothermia were ineffective in clinical trials [14]. So far, injury prevention remains the most efficient way for controlling TBI. Furthermore, it seems unlikely that any treatment aimed at injury reversal will be as effective as injury prevention. The preventive measures against bTBI include the use of personal protective equipment such as Kevlar vests or helmets. Even with an advanced helmet design, a significant part of the head remains exposed to the blast. The prevalent incidence of bTBI in recent military conflicts and the frequency of sports-related concussions indicate that the effectiveness of personal protective equipment against bTBI is insufficient [7, 15, 16].

Recently, it was suggested that increasing ICP and reducing slosh by compression of the internal jugular vein (IJV) markedly reduced markers of neurological injury in acceleration- deceleration TBI models [17, 18]. This finding suggests that the development of a neck collar as a potential protective tool against closed head TBI might be possible. The precise underlying mechanisms of bTBI, as well as the neuropathological, pathophysiological, and functional consequences of bTBI, remain unknown.

In the current study, we tested whether IJV compression could provide protection of brain tissue against blast-induced traumatic brain injury. It is still unknown if exposure to BOP or rapid acceleration due to impact is the leading transmission mode of injury [19]. A recent study in a mouse model by Gullotti et al. [20] reported that restricting the movement of the head significantly increased survivability and decreased cognitive deficits following blast exposure. Head immobilization was most effective against parallel exposure to the blast wave as opposed to a perpendicular orientation. In our study, we minimized linear/rotational head movement by head restriction and by exposing animal in the frontal (face-on) orientation to blast.

## 2. Materials and Methods

### 2.1. Animals and Exposure to BOP

Male Sprague-Dawley (SD) rats weighing 325–400 g (Taconic Farm, Hudson, NY) were used in the study. Immediately before exposure to BOP, animals were anesthetized with 5% isoflurane, secured in a holder placed inside a compressed air-driven shock tube, and subjected to BOP in frontal (face-on) orientation. The animals were restrained in the holder to ensure adequate immobilization during blast exposure. The animals were exposed to three 110 ± 5 kPa exposures separated by 30 min and were allowed to recover from anesthesia after each exposure. The reference pressure outside the animal was measured by two piezoelectric sensors (PCB Piezotronics Inc., Buffalo, NY) placed on opposite sides at the level of rat's head. One sensor was aligned parallel to the direction of propagation of BOP, measuring static pressure, while the other sensor was aligned perpendicular to the blast flow to measure reflected pressure. The sensors were connected to a signal conditioning unit which provided input to PowerLab and LabChart (AD Instruments, Colorado Springs, CO) for data acquisition (collection rate 100 kHz), display, and recording [21]. The values, expressed as average (standard error) for *n* = 6, of peak amplitude, duration, and impulse for positive excursion of static pressure (overpressure) were 110 (5.20) kPa, 7.82 (0.061) ms, and 356.93 (2.28) kPa·ms, respectively. The average (*n* = 6) parameters of reflected pressure were peak pressure of 148.9 (0.73) kPa, overpressure duration of 7.93 (0.021) ms, and overpressure impulse of 615.338 (4.67) kPa·ms.

### 2.2. IJV Compression

The blood compartment is a single intracranial compartment that can be easily subjected to rapid changes in volume and pressure resulting in variation in the intracranial compliance. Mechanical obstruction of the veins in the neck inhibits the outflow of blood from the brain and increases ICP [17]. Mechanical obstruction by IJV compression was applied by tightening Velcro tape around the animal's neck [17] without compromising the airway. The tape was placed on the rat's neck for approximately one minute before BOP exposure. Immediately after exposure, the tape was removed from animals. The increase in ICP occurred within seconds following IJV compression. The parameters of compression were determined in a separate group of animals (*n* = 5) which were not used for histopathology as an additional surgical procedure could interfere with neuropathological assessment. In that group an increase in ICP concurrent with IJV compression was confirmed by measuring ICP with a FISO pressure probe (FISO Technologies, Quebec City, Canada) which was placed in the lateral ventricle, as previously described [21]. The baseline ICP was 4.9 ± 0.4 mmHg and was increased to 12.5 ± 2.4 mmHg following IJV compression (*p* < 0.01).

### 2.3. Animal Grouping

Animals were randomly assigned to three groups (*n* = 6 in each group): one control group (Con) without exposure to BOP and two blast groups: one group without IJV compression (Blast) and a second group with IJV compression (I-Blast). All groups were treated identically including receiving anesthesia and being placed in the blast tube. One day following exposure, the rats were euthanized with Euthasol (Virbac AH, Inc., Fort Worth, TX) and brain tissues were removed and fixed with 10% formalin.

### 2.4. Histology and Immunohistochemistry

Brain tissues fixed in 10% formalin were embedded in paraffin. Coronal sections (5 *μ*m) were prepared from the frontal cortex (1.8 mm anterior and 2.8 mm posterior to Bregma) to the hippocampus (2.3 mm posterior and 4.3 mm posterior to Bregma) and stained with hematoxylin-eosin (H&E). Sections were assessed for neuronal morphological changes with a Nikon light microscope equipped with a CCD Spot camera.

Neuronal marker, neuronal nuclei (NeuN), water channel membrane protein, aquaporin-4 (AQP-4), oxidative/nitrosative marker, 3-nitrotyrosine (3-NT), and vascular marker, endothelin 1 receptor A (ETRA), were analyzed to measure brain damage after exposure to BOP. Brain sections were incubated with mouse anti-NeuN (1 : 1000, Millipore), rabbit anti-AQP-4 (1 : 1000, Invitrogen), rabbit anti-nitrotyrosine (1 : 200, Millipore), and rabbit anti ETRA (1 : 500, Sigma) overnight at 4°C. After washes in PBS, the sections were incubated with the appropriate secondary antibodies labeled with Cy2 or Cy3 (1 : 500, Jackson ImmunoResearch) for 2 h at room temperature or overnight at 4°C. Slides were examined with a Nikon fluorescence microscope and images were captured with a CCD Spot camera.

### 2.5. Immunofluorescence Quantification

Images of six to eight sections were taken from each specified brain area per animal and were analyzed using Pro Image J Plus software. The average density of immunofluorescence of each animal was calculated based on the values of density in six to eight sections and was expressed as mean ± SEM. The statistical significance between groups was compared by ANOVA followed by Fisher LSD test for multiple comparisons.

## 3. Results

### 3.1. Histopathological Changes in Cortex and Hippocampus Exposed to BOP

**Figure 1:**
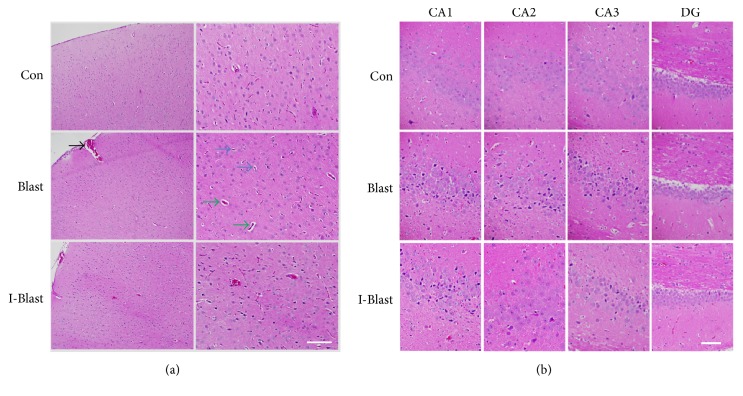
Representative photomicrographs of coronal paraffin sections stained with H&E. (a) Signs of cytoplasmic vacuolization and nucleus shrinkage (blue arrows) and perivascular vacuolization (green arrows) were observed in cortex of rats exposed to BOP. Presence of subarachnoid space hemorrhage (black arrow). (b) Histopathologic morphology in hippocampus of rats exposed to BOP. Intense eosinophilia of the cytoplasm is found in CA1, CA2, and CA3 regions but not Dentate Gyrus (DG) of hippocampus in brain after exposure to BOP. Scale bars: 200 *μ*m (a) and 100 *μ*m (b). Con, nonexposed control; Blast, animals exposed to blast; and I-Blast, animals with IJV compression exposed to BOP.

H&E staining demonstrated some micropathological changes in frontal cortex and hippocampus one day after exposure to blast (Figures 1(a) and 1(b)). The pattern of brain damage corresponds with the detailed analysis of brain histopathology previously described in the same blast model [22].

### 3.2. Effect of Exposure to BOP on Neuronal Loss

**Figure 2:**
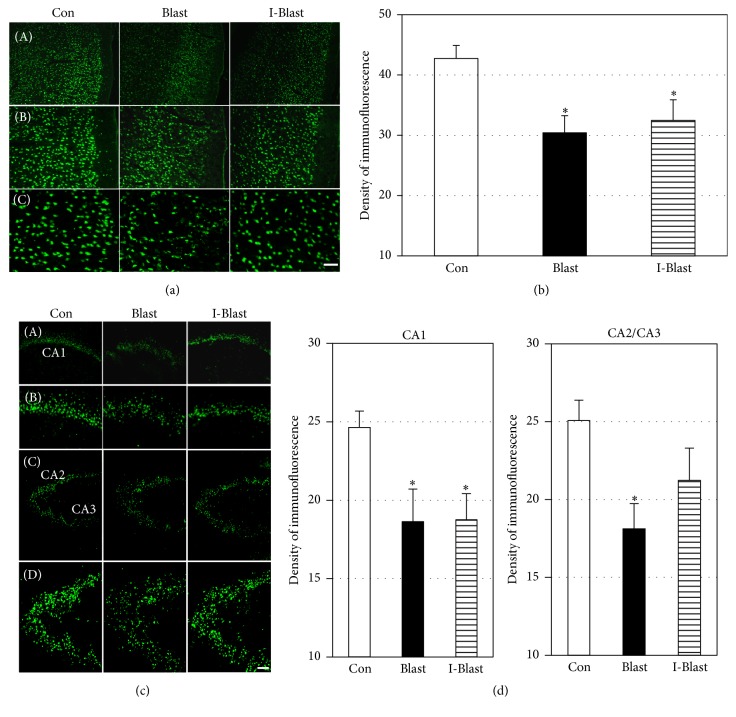
(a) Microphotographs of NeuN immunoreactivity in frontal cortex. Scale bars: 200 *μ*m (A), 100 *μ*m (B), and 50 *μ*m (C). (b) Quantitative analysis of immunofluorescence density of NeuN in frontal cortex. Density of fluorescence is significantly decreased in both blast groups compared with controls (Con). ^*∗*^*p* < 0.05 (*n* = 6). (c) Microphotographs of NeuN immunoreactivity with low and high magnifications in CA1, CA2, and CA3 regions of hippocampus. Scale bars: 200 *μ*m ((A) and (C)) and 100 *μ*m ((B) and (D)). (d) Quantitative analysis of NeuN immunofluorescence density in CA1, CA2, and CA3 regions of hippocampus. Density of fluorescence is significantly decreased in CA1 in both BOP groups. The blast-induced decrease of NeuN immunoreactivity in CA2 and CA3 regions is ameliorated with IJV compression (I-Blast). ^*∗*^*p* < 0.05, control versus blast exposed animals (*n* = 6/group). Note that *x*-axes in (c) and (d) do not cross *y*-axis at zero density.

In order to further examine the effect of BOP on neuropathological damage in cortex and hippocampus, immunoreactivity of mature neuronal marker, NeuN, was assessed after BOP exposure. NeuN-staining showed a significant decrease of immunoreactive neurons in cortex in both BOP groups by ~30% compared with controls (Figures 2(a) and 2(b)). In hippocampus, NeuN immunoreactivity in the CA1 region was reduced by 30% compared with nonexposed controls, with no significant differences between the two BOP exposed groups (Figures 2(c) and 2(d)). In the CA2 and CA3 regions of hippocampus, a significant decrease of NeuN immunoreactivity was observed only in one blast group (Blast) (Figures 2(c) and 2(d)). IJV compression attenuated the BOP-induced decrease of NeuN immunoreactivity in CA2/CA3 areas to a level not significantly different from controls (Figures 2(c) and 2(d)).

### 3.3. Effect of BOP on Brain Cytotoxic Edema

**Figure 3:**
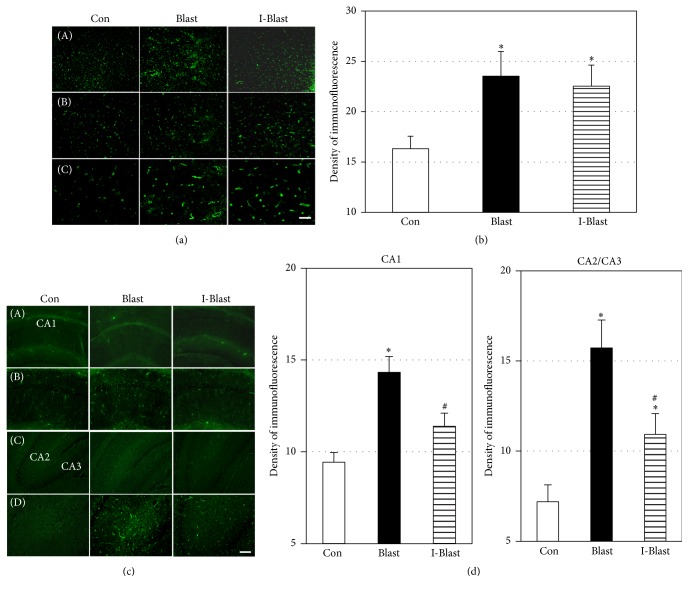
(a) Microphotographs of AQP-4 immunoreactivity in frontal cortex. Scale bars: 200 *μ*m (A), 100 *μ*m (B), and 50 *μ*m (C). (b) Quantitative analysis of AQP-4 fluorescence density in cortex. The density of fluorescence is significantly increased in cortex of both BOP exposed groups compared with controls. ^*∗*^*p* < 0.05 (*n* = 6/group). (c) Microphotographs of AQP-4 immunoreactivity with low and high magnifications in CA1, CA2, and CA3 regions of rat hippocampus. Scale bars: 200 *μ*m ((A) and (C)) and 100 *μ*m ((B) and (D)). (d) Quantitative analysis of AQP-4 fluorescence density in CA1, CA2, and CA3 regions of hippocampus. The density of fluorescence is significantly increased only in CA1 region of the blast exposed rats (Blast). IJV compression significantly attenuated the increase (I-Blast). In CA2 and CA3 regions, IJV compression (I-Blast) significantly reduced increase of AQP-4 immunoreactivity compared with the Blast group. ^*∗*^*p* < 0.05, control versus Blast group (*n* = 6/group); ^#^*p* < 0.05, I-Blast versus Blast group (*n* = 6/group). Note that *x*-axes in (c) and (d) do not cross *y*-axis at zero density.

The upregulation of AQP-4, a water-channel protein, has been shown to facilitate cerebral cytotoxic edema formation in several TBI models [22–24]. After exposure to BOP, AQP-4 expression was significantly increased in cortex of both BOP exposed groups compared with the control group (Figures 3(a) and 3(b)). In hippocampus, AQP-4 expression was increased after BOP across all areas, CA1 and CA2/CA3. IJV compression significantly eliminated or reduced this increase (Figures 3(c) and 3(d)).

### 3.4. Effect of BOP on Oxidative/Nitrosative Stress

**Figure 4:**
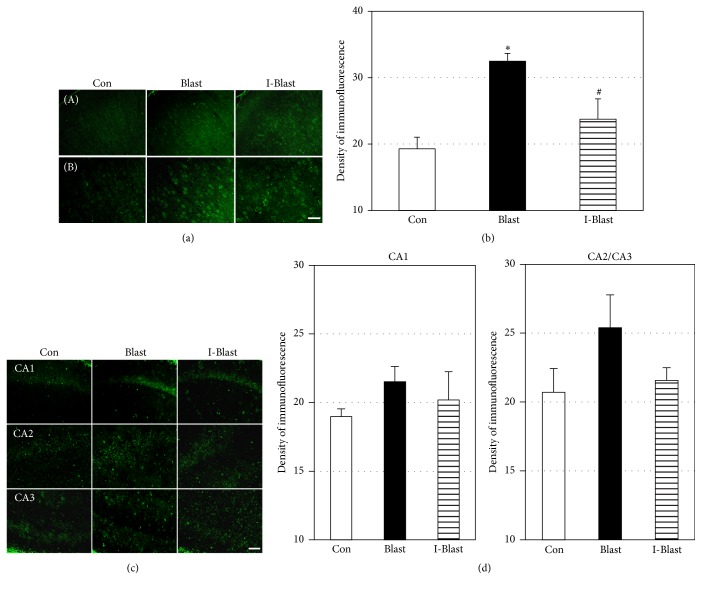
(a) Microphotographs of 3-NT immunoreactivity in frontal cortex. Scale bars: 100 *μ*m (A) and 50 *μ*m (B). (b) Quantitative analysis of fluorescence density of 3-NT in cortex. The blast-induced increase of 3-NT is prevented by IJV compression. Con, nonexposed control; Blast, animals exposed to blast; and I-Blast, animals with IJV compression exposed to BOP. ^*∗*^*p* < 0.05, control versus Blast (*n* = 6/group); ^#^*p* < 0.05, I-Blast versus Blast (*n* = 6/group). (c) Microphotographs of 3-NT immunoreactivity in CA1, CA2, and CA3 regions of rat hippocampus. Scale bar: 50 *μ*m. (d) Quantitative analysis of fluorescence density of 3-NT in CA1, CA2, and CA3 regions of hippocampus. There was no significant difference of fluorescence density among experimental groups (*n* = 6/group). Note that *x*-axes in (c) and (d) do not cross *y*-axis at zero density.

Oxidative stress is known to play a role in the secondary injury mechanism following TBI [10–12] and 3-NT is one of the markers of oxidative/nitrosative damage. Immunohistochemical quantification of 3-NT was performed to determine the extent of the oxidative mechanism activation after BOP in cortex and hippocampus. Over 50% increase in 3-NT immunoreactivity was observed in the cortex of the blast group (Blast). IJV compression (I-Blast) significantly attenuated this increase to a level that was not significantly different from the control group (Figures 4(a) and 4(b)). In hippocampus, BOP did not significantly activate 3-NT expression in CA1, CA2, and CA3 areas (Figures 4(c) and 4(d)).

### 3.5. Effect of BOP on ETRA

**Figure 5:**
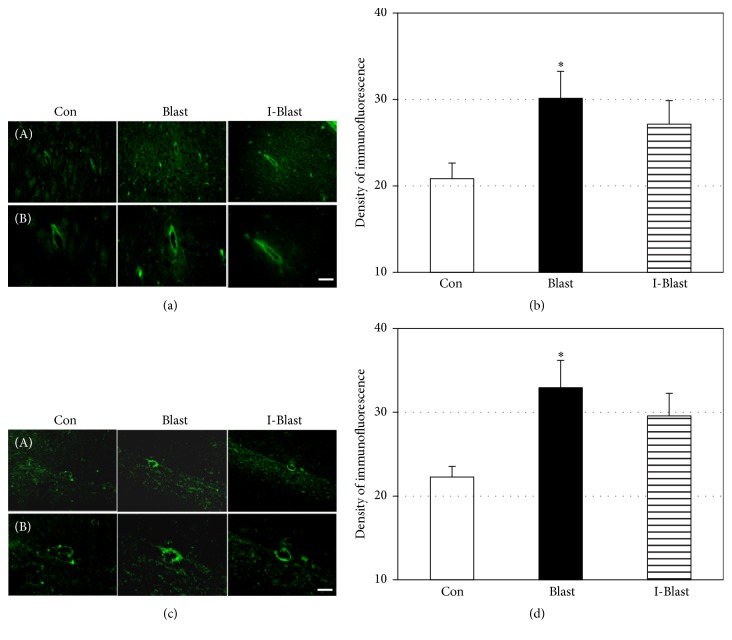
(a) Microphotographs of ETRA immunoreactivity in frontal cortex. Scale bars: 100 *μ*m (A) and 50 *μ*m (B). (b) Quantitative analysis of fluorescence density of ETRA in cortex. The density of fluorescence is significantly increased in cortex of rats after blast. IJV compression did not significantly change the blast-induced increase in ETRA. Con, nonexposed control; Blast, animals exposed to blast; and I-Blast, animals with IJV compression exposed to BOP. ^*∗*^*p* < 0.05, control versus Blast (*n* = 6). (c) Microphotographs of ETRA immunoreactivity in hippocampus. Scale bars: 100 *μ*m (A) and 50 *μ*m (B). (d) Quantitative analysis of fluorescence density of ETRA in hippocampus. The density of ETRA fluorescence was significantly increased after blast and remained elevated after IJV compression, ~25% over controls. ^*∗*^*p* < 0.05 compared with control (*n* = 6/group). Note that *x*-axes in (c) and (d) do not cross *y*-axis at zero density.

Cerebral vessels could be directly damaged by oxidants or by inflammation [25]. Endothelin 1 receptor A, ETRA, is localized in cerebral vessels and has been reported to be upregulated after TBI [26–29]. The upregulation of ETRA is a component of cerebral microvessel alterations and vascular remodeling in TBI [27, 30]. In order to determine the vulnerability of cerebral vasculature to BOP, immunoreactivity of ETRA was assessed in brains of BOP exposed animals. The immunoreactivity of ETRA was significantly increased by over 50% in the cortex and hippocampus of the blast group. There was a nonsignificant difference between two blast exposed groups, Blast and I-Blast, in ETRA expression (Figures 5(a) and 5(b)).

## 4. Discussion

Several mechanisms of bTBI have been suggested; however, their relative contribution to brain damage remains unclear. These mechanisms include (1) direct interaction of the pressure wave with skull and subsequent transmission of energy through the brain, (2) transfer of kinetic energy from the blast wave through either the vascular system or cerebrospinal fluid, and (3) some indirect mechanism(s) caused by systemic factors and damage to other organs and tissues [31]. These proposed mechanisms are most likely not mutually exclusive and their contributions to brain damage can differ in various situations depending on intensity and spatial orientation to blast as well as on the presence of head and/or body protection.

The most effective protection would eliminate interaction between blast waves and brain with mechanical protective equipment, such as helmets and body shields. The emphasis on helmet technology has been only partially successful given the limitations of helmets to block acceleration-deceleration mechanisms which result in brain concussions [17]. In addition, helmets do not protect all skull areas and make them vulnerable to blast wave penetration. Recently, it was demonstrated that reduction in intracranial compliance by increased intracranial volume induced by IJV compression reduced brain injury caused by mechanical weight impact [18]. This effect is known as “slosh” and it is defined as the fluid dynamic forces that cause movement of the brain inside the cranium [32]. It has been suggested that mitigating “slosh” reduces differential motion and collision between brain and skull and ameliorates brain damage observed in a model of weight impact TBI [18]. The increases in the volume of fluid in the skull can be achieved by altering the volume of blood and CSF. Compression of brain venous outflow is the simplest way to increase brain fluid volume; as the inflow of arterial blood continues, the venous pressure increases along with an increase in ICP.

It is not known if the same mechanism of protection against cortical weight impact TBI by IJV compression can be as efficient against bTBI. TBI, as a result of mechanical impact on brain, is in large extent caused by acceleration-deceleration mechanisms with brain movements against the cranial wall and concussions being the predominant form of injury. During exposure to BOP, the primary damaging mechanism involves transfer of the shock wave energy through the skull and its interaction with brain tissue, while the damage by acceleration-deceleration mechanisms depends on the orientation to blast [33, 34]. More recent studies [8, 35–38] have supported the theory that the pathophysiological mechanism of blast TBI also involves damage to the BBB and tiny cerebral blood vessels, which is caused by blood surging quickly through large blood vessels from the torso to the brain [39]. The direct mechanism of the primary bTBI also includes skull flexure [40, 41]. Results from numerical hydrodynamic simulations suggest that nonlethal blasts can induce sufficient skull flexure to generate potentially damaging loads in the brain, even without a head impact [40]. Skull flexure is also thought to be a contributing factor to the pressure wave measured inside the brain following exposure to BOP in both rats and swine depending on orientation to BOP, location of pressure probes, and skull thickness [42–44].

In the current study, we assessed histopathological signs of blast-induced brain damage and the possible mitigating effect of IVP by immunofluorescence staining of specific markers of neuronal loss, brain edema, stress of oxidation/nitration, and vascular dysfunction. Immunofluorescence of neuronal marker NeuN was significantly decreased and expression of AQP-4 and ETRA was upregulated in both cortex and hippocampus consistent with neuronal loss, edema formation, and vascular dysfunction following exposure to BOP. The only exception was that 3-NT was increased in cortex but not in hippocampus. Consistent with these results, similar pathological changes induced by single or repeated exposure to 120 kPa BOP were previously reported [22, 45, 46]. These changes were seen as early as 3 h after blast and persisted for at least two days after BOP exposure. Furthermore, a selective vascular pathology and damage of BBB integrity were characteristic signs of bTBI [23, 47]. It has been suggested that a cascade of reactions initiated in the early phase after exposure to BOP, such as activation of oxidative mechanisms of damage, evolve into dysfunction of the BBB and damage to the vascular endothelium followed by impaired blood flow, neuronal apoptosis, and inflammatory damage to the brain [48]. The BOP-induced cellular pathology may be selective in different brain regions with different time courses of manifestation after blast exposure. This possibility was supported by the recent study demonstrating more extensive damage to the BBB integrity in hippocampus than in other brain regions [49]. On the other hand, more neuronal degeneration was observed in prefrontal cortex than in hippocampus at seven days after exposure to BOP [50].

It should be noted that our protocol minimized any contribution of acceleration-deceleration mechanism of damage by head movement, since the animal's head was fixed to a solid holder and animals were exposed in the frontal, head-on position to the direction of BOP. Based on our results in the Blast group, it appears that, even with elimination of the head movement and acceleration-deceleration mechanisms, other mechanisms such as skull flexure and blood surge may still generate pressure gradient and brain damage after BOP.

The aim of this study was to determine if IJV compression, a maneuver designed to reduce cerebral compliance and increase ICP, can protect against blast-induced TBI. We did not instrument our animals to collect hemodynamic data. However, a study by Chou et al. [51] demonstrated that manual IJV compression in rats decreased venous return and increased ICP, while mean arterial pressure (MAP) decreased. The drop in MAP and increase in ICP may potentially result in reduced cerebral perfusion pressure (CPP) that is defined as a difference between MAP and ICP. During 1 minute of manual jugular vein compression, the ICP increased from 6.9 to 9.2 mmHg and MAP decreased from 97 to 90 mmHg, resulting in a calculated CPP decrease from 90 mmHg to 81 mmHg during the test. This value remains above the critical CPP at which cerebral blood flow begins to fall (50 mmHg). Within 30 s after release of compression, both ICP and MAP returned to their control values. Based on their results, it appears that IJV compression for 1 min does not result in the CPP falling below the critical level.

The protective effect of IJV compression was evaluated in frontal cortex and hippocampus as areas predominantly involved in cognition, learning and memory, and emotional functions that are related to behavioral impairment after BOP [46, 52–54]. Our data indicate that IJV compression reduced selective pathological changes after exposure to BOP. IJV compression appeared to be more effective in attenuating the NeuN decrease and the upregulation of AQP-4 in hippocampus as compared to the cortex, suggesting a selective effect of IJV compression. On the other hand, the extent of oxidative and nitrosative damage as determined by 3-NT immunoreactivity was higher in cortex and IJV significantly prevented the 3-NT increase. ETRA fluorescence was elevated in both the cortex and the hippocampus after BOP and remained elevated after IJV compression around 25% over control. Our results show that the decrease in brain compliance by increase in ICP can alleviate brain damage not only in the blunt trauma induced TBI but also in bTBI, in this case likely by mitigating the contribution of skull flexure to the interaction of brain with the cranium.

Plastic neck collars have already been developed to mildly compress the jugular vein bilaterally to increase intracranial blood volume [55]. The collar was worn by hockey players at each practice session and game over the entire hockey season to prevent brain concussions by mechanical impact. The collar device did not cause any structural brain damage or change in cerebral metabolism as evidenced by MR imaging and EEG recording. It was found that the group of players that wore the collars had reduced alterations in white matter compared with the noncollar group.

In conclusion, the present study indicates that IJV compression has a protective potential in bTBI by attenuating blast-induced neurodegeneration, edema, oxidative damage, and vascular dysfunction. Although further studies are needed to explore the mechanism(s) mediated by slosh mitigation and its potential clinical application, our findings suggest that IJV compression has significant promise as a protective measure against bTBI.
